# Modelling Food and Population Dynamics in Honey Bee Colonies

**DOI:** 10.1371/journal.pone.0059084

**Published:** 2013-05-07

**Authors:** David S. Khoury, Andrew B. Barron, Mary R. Myerscough

**Affiliations:** 1 School of Mathematics and Statistics, The University of Sydney, Sydney, New South Wales, Australia; 2 Department of Biological Sciences, Macquarie University, Sydney, New South Wales, Australia; 3 Centre for Mathematical Biology, The University of Sydney, Sydney, New South Wales, Australia; Monash University, Australia

## Abstract

Honey bees (*Apis mellifera*) are increasingly in demand as pollinators for various key agricultural food crops, but globally honey bee populations are in decline, and honey bee colony failure rates have increased. This scenario highlights a need to understand the conditions in which colonies flourish and in which colonies fail. To aid this investigation we present a compartment model of bee population dynamics to explore how food availability and bee death rates interact to determine colony growth and development. Our model uses simple differential equations to represent the transitions of eggs laid by the queen to brood, then hive bees and finally forager bees, and the process of social inhibition that regulates the rate at which hive bees begin to forage. We assume that food availability can influence both the number of brood successfully reared to adulthood and the rate at which bees transition from hive duties to foraging. The model predicts complex interactions between food availability and forager death rates in shaping colony fate. Low death rates and high food availability results in stable bee populations at equilibrium (with population size strongly determined by forager death rate) but consistently increasing food reserves. At higher death rates food stores in a colony settle at a finite equilibrium reflecting the balance of food collection and food use. When forager death rates exceed a critical threshold the colony fails but residual food remains. Our model presents a simple mathematical framework for exploring the interactions of food and forager mortality on colony fate, and provides the mathematical basis for more involved simulation models of hive performance.

## Introduction

A honey bee colony gathers dispersed floral resources (pollen and nectar) from the environment to a central place, and processes them to provide food to support the current population and rearing of the next cycles of brood. Previously we proposed a simple mathematical model of honey bee population dynamics to explore the impact of varying forager death rate on colony growth and development [1]. This model was a deliberate simplification to consider how interactions between adult foragers and hive bees and brood might influence colony growth. However in natural colonies food availability may impose limits on colony development. Here we present a new model to explore how changes in food availability might interact with behavioural and social processes in the colony to influence colony growth.

This issue is pertinent because the amount of honey that can be extracted from commercial bee hives for human use depends on bees collecting nectar in excess of what is needed to support their population, and storing the excess as honey. The honey industry is therefore reliant on manipulating the flux of food through a colony to maximize the excess, and understanding the relationship between food availability and colony growth may improve colony management practice. Further, recent concerns about the sustainability of bee populations [2] have highlighted a need to better understand how healthy colonies function, and why they may sometimes fail.

All the nutritional demands of a honey bee colony are met by supplies of pollen and nectar gathered by foragers [3]. Nectar is entirely carbohydrate in the form of simple sugars (with sometimes some trace minerals and allelochemicals) [4], [5]. Pollen provides bees with lipids, protein, and vitamin and mineral nutrients [3], [5]. Nectar is transferred from foragers to non-foraging hive bees who deposit the nectar in cells and, over time, process and concentrate it to form honey [5]. Pollen is deposited directly in cells by foragers, but mixed with a small amount of nectar and packed by hive bees for storage [5]. Honey, nectar and pollen are consumed by hive bees and used to produce a protein rich brood food, which is fed to the queen and developing larvae [3], [5]. In seasonal climates during winter the colony relies on stored pollen and nectar collected over the summer [5].

Honey bees have a very typical pattern of age polyethism performing various functions within the hive for the first two to three weeks of their adult life before transitioning to foraging [5], but bee behaviour is very sensitive to changes in levels of stored food or food influx. A shortage of food within the colony stimulates a precocious onset of foraging in adult bees truncating the amount of time they spend as hive bees [6], [7]. Brood rearing is especially sensitive to food shortages [3]. Typically a colony does not maintain a large store of pollen, and interruptions in pollen inflow to a colony can trigger cannibalism of developing larvae by worker bees [8], [9], [10]. This is interpreted as an adaptive response by workers to reduce the size of the brood population to that most likely able to be successfully reared when food is limited [3], [9].

The interactions between food and population dynamics in a bee colony are therefore quite complex. Food collection is influenced by the size of the forager population, and in turn food flux through the colony can influence the size of the forager population by altering the rate at which hive bees become foragers and the size of the brood population, which will eventually become the next generation of foragers. The model we present here offers a simple theoretical framework with which to explore how the dynamics of food flow through a colony might interact with population dynamics to determine colony growth.

## Methods

### Constructing a demographic model which includes brood and food dynamics

In Khoury *et al.*, [1] we constructed a model for the population of a hive of honey bees which only included the adult bees. These were divided into two classes; hive bees and foragers. We assumed that the rate that adults emerged from pupation was a function of hive size only and that food was not a limiting factor in hive population dynamics. We also assumed that the hive had sufficient available food so that food scarcity did not affect the population dynamics.

**Figure 1:**
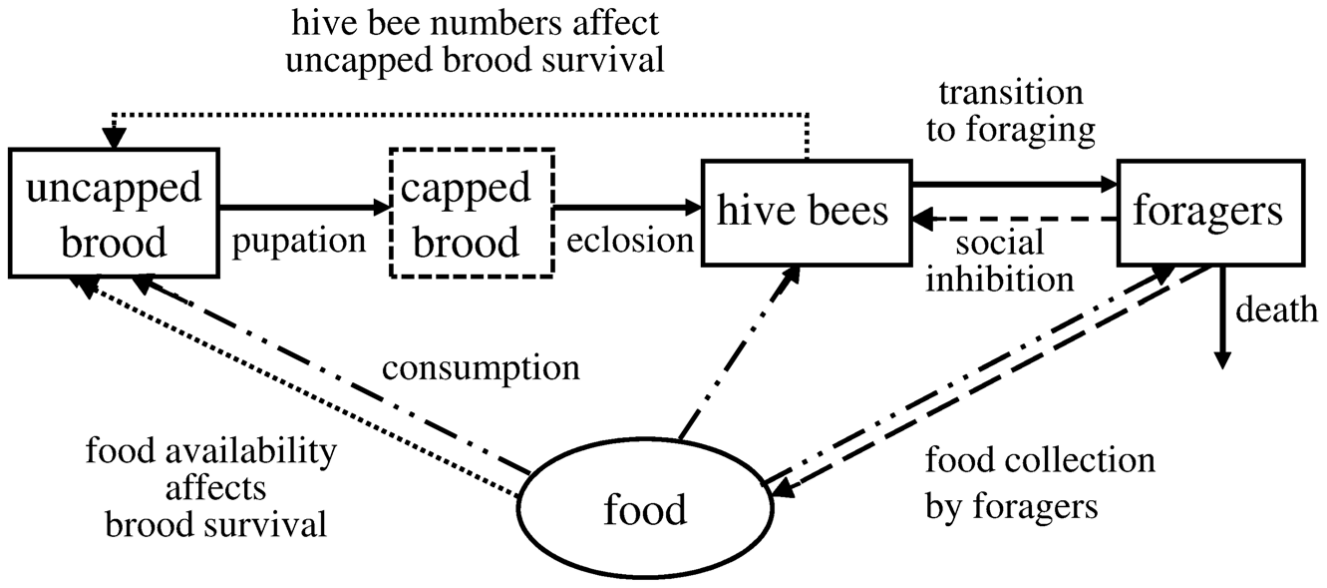
Honey bee social dynamics represented in the model. The dash-dot lines represent consumption of food. The class “capped brood” appears in a box with a dashed border because it is not explicitly modeled although the period that brood spends as capped pupae is accounted for by the delay 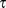 in the equations.

Here we extend the model of Khoury *et al.*, to include both food and brood explicitly (Figure 1). As before, we only consider the population of female worker bees since it is only females that contribute to foraging and colony maintenance. Let *B* be the number of uncapped brood in the hive, *H* be the number of hive bees and *F* the number of foragers. Let *f* be a measure of the amount of food that is stored in the hive and available for the colony to use. We do not distinguish between pollen and nectar (protein and carbohydrates) here. Our aim is to keep the model simple so that we can perform comprehensive analyses and model gross effects transparently. We assume that the survival of uncapped brood (eggs and larvae) is dependent on the number of hive bees available to tend and feed brood, on food availability and on the laying rate *L* of the queen. Larvae become pupae inside cells that are capped by worker bees and we assume that pupation occurs at a constant rate proportional to the amount of brood present. This is a simplifying assumption that allows us to continue to use a compartment model rather than a more complicated model with explicit age structure. Adult bees emerge 12 days after pupation and we assume that mortality of capped brood is negligible. We also assume that the death rate of hive bees is negligible. Foragers are recruited from the hive bee class and die at a rate *m*. Let *t* be the time in days. Then we can represent the model illustrated in Figure 1 as four differential equations:

Rate of change of brood numbers:
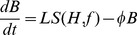
(1)


The first term represents laying and survival of brood where *L* is the laying rate of the queen and *S(H,f)* is a function of food and hive bee numbers. We assume that *S(H,f)* becomes constant as *f* and *H* become large and that the dependence of food and hive bee numbers is independent of one another. With these assumptions, we can model *S(H,f)* as
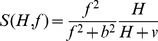
(2)where *b* and *v* are parameters that determine how rapidly *S(H,f)* tends to one as *f* and *H* increase respectively. The first term in *S(H,f)* models the way that brood survival declines when food stores are low. This decline in brood survival has two causes: brood die because there is not enough food to feed them as they develop; and because workers cannibalise the eggs and young larvae when food is scarce to recycle protein in the colony and so increase the likelihood of older larvae surviving to pupation. We assume that when food levels are very low there is almost no survival, but that survival rates climb rapidly when food reaches a viable level. Consequently we use a sigmoid form for this term. The second term models the impact of hive bee numbers on brood survival. When there are few hive bees there may be insufficient workers to supply all the larvae with the food that they need, even if the hive has large food stores. Also, low hive bee numbers will impact on the colony's ability to keep the brood warm so that they develop properly [11]. We assume that when hive bee numbers are low the amount of brood that is raised is close to a linear function of hive bee numbers and so we choose a Michaelis-Menten type of function for this term.

Rate of change of hive bees:
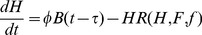
(3)where 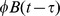 is the rate that adult bees emerge from pupation. The bees that are emerging at time *t* are the same bees that entered pupation at 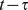. The function *R(H,F,f)* gives the proportional rate that hive bees make the transition into foragers. This rate is a function of hive bee and forager numbers, but also depends on stored food supply.

Rate of change of foragers:
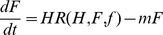
(4)where the first term is the rate that hive bees become foragers and the last term is the rate that foragers die.

We assume that the transition from hive bee to forager has an underlying component that is increased by the absence of stored food and reduced by social inhibition due to the presence of foragers in the hive. We write the recruitment function as
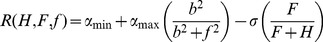
(5)where 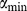 is the rate that hive bees become foragers when there is plenty of stored food but no foragers in the hive, 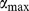 governs the strength of the effect that low food stores have on the transition to foragers and *b* controls the rate that the food-dependent terms decrease as food stores increase. Social inhibition depends on the proportion of foragers in the adult bee population, and the strength of this inhibition is governed by 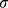. The forager-to-hive-bee transition depends on food stores in a similar way to how brood survival depends on food stores as both share the same parameter *b*. This implicitly assumes that shortage of food for the larvae is one of the stimuli that drive increased forager recruitment.

Rate of change in food stores:
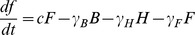
(6)where *c* is the average amount of food collected per forager per day. In reality, this will vary seasonally, daily or even hourly, but for the current purposes we will assume that it is constant. The consumption of stored food by brood, hive bees and foragers is given by 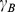, 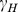 and 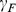 respectively. Because the ratio of foragers to hive bees in the hive quickly equilibrates we will assume that we can model food consumption by adult bees using the average consumption 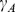 so that equation (6) can be written as
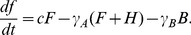
(7)


#### Solution and analysis of the model

Differential equations (1), (3), (4) and (7), using functions (2) and (5), were solved using MATLAB to get typical plots of brood, hive bees, foragers and food against time. When there are very large stores of food (that is as f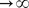) then, to a good approximation
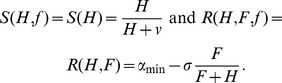
(8)


In other words, brood survival and transition to foraging do not depend on the size of the food stores in the hive; there is easily enough food to provide for all the hive's needs. We solved for the steady state of the model, both when food is present in abundance and when food is limited, and we obtained a solution for the value of *m* where the hive changes from having abundant food to being food-limited. We also found the value of m*, the critical death rate, where the hive goes extinct. These are all given in Appendix S1.

#### Choosing parameters for the model

Following Khoury et al., [1], we set the queen's daily egg laying rate at *L* = 2000, the transition rate in the absence of foragers but in the presence of food to 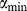 = 0.25 and the parameter governing social inhibition of forager recruitment to 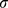 = 0.75. These values assume that the youngest age at which a worker can become a forager is 4 days old [12] and that reversion of foragers to hive bees can only begin if more than one third of the worker bees are foraging [13]. We set the parameter which controls the effect of the hive bees on brood survival at *v* = 5000. This assumes that when there are 5000 hive bees, roughly half the eggs that are laid fail to survive to pupation because of lack of attention by worker bees, which is congruent with our observations of experimental colonies, and above the minimum population size at which colonies can effectively rear brood [14], [15]. We chose 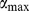 = 0.25 so that the recruitment of hive bees in the absence of foragers was doubled when food was absent as well [7]. We set the pupation rate of brood to 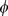 = 1/9, which assumes that it takes 9 days from the time that an egg is laid until the larvae pupates and we set the duration of pupation 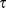 to 12 days.

We measured food in units of grams. Using data from Harbo [16], [17], Russell et al [18] estimate that foragers collect approximately 0.1 grams of food per forager per day, when food is plentiful in the environment. Other data from Harbo [17] suggests that it requires about 0.163 g of honey to rear a worker bee to the point of pupation, so if we average this over the nine days that brood is uncapped we get that the consumption rate per brood item 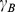 = 0.018 g/day. In reality food is consumed only by larvae, but we have not separated eggs from larvae in this model and so take an average amount over the whole period before pupation. The rate of honey consumption for adult bees is given as 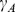 = 0.007 [17]. If we assume that the effects of low food stores are not evident when there is a kilogram or more stored food then we can estimate that *b* = 500 so that *b^2^*/(*b^2^*+*f^2^*) = *f^2^*/(*b^2^*+*f^2^*) = ½ when *f* = 500, that is when there is 500 g of stored food.

## Results

**Figure 2:**
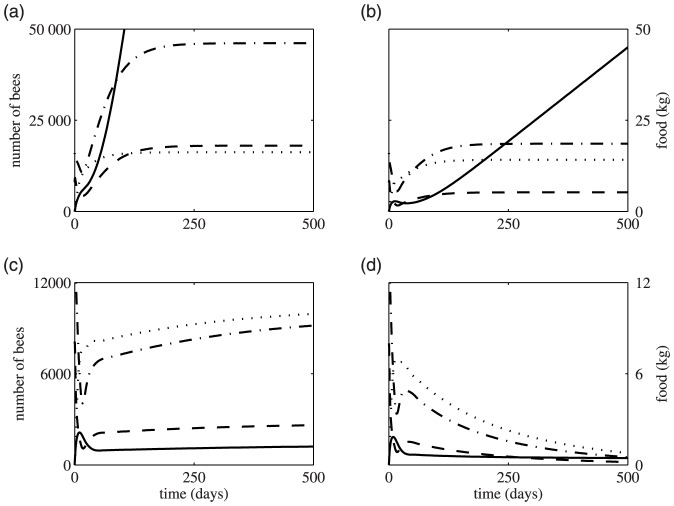
Population and food behaviour over time for different rates of forager mortality. In all plots, the solid line represents food, the dashed line foragers, the dash-dot line hive bees and the dotted line brood. Parameter values are *L* = 2000, 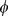 = 1/9, *v* = 5000, 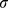 = 0.75, 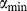 = 0.25, 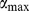 = 0.25, *b* = 500, *c* = 0.1, 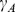 = 0.007, 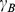 = 0.018, and 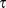 = 12. In (*a*) *m* = 0.1; (*b*) *m* = 0.3;(*c*) *m* = 0.42; (*d*) *m* = 0.5. In all plots the hive starts with 16000 hive bees, 8000 foragers and no brood or food at *t* = 0. Note that (*c*) and (*d*) have a different vertical scale to (*a*) and (*b*).

Figure 2 shows brood, hive bees, forager and food stores as a function of time for increasing death rate *m* with all other parameters held constant. When death rates are low (*m* = 0.1, Figure 2a), food stores rise rapidly while the bee populations tend to a steady state that is essentially determined by the balance between the death rate of foragers and the laying rate of the queen. At a higher death rate (*m* = 0.3, Figure 2b), the rate of food accumulation is decreased, and the equilibrium population size is reduced. At even higher death rates (illustrated here by *m* = 0.42, Figure 2c) food becomes a limiting factor as the hive does not have enough foragers to collect more food than it consumes, so that the amount of stored food does not continually increase but settles at a steady state. Bee populations are also much lower. For death rates above the critical death rate, m* the bee population goes extinct but stored food remains in the hive, even after all bees have died (Figure 2d). This is probably because the hive bee population and hence the brood rearing effort are so compromised that the population declines faster than residual food stores can be consumed.

**Figure 3:**
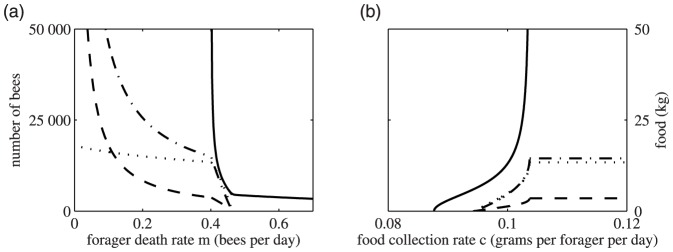
Steady state population as a function of (a) death rate and (b) food collection rate. Parameter values are *L* = 2000, 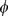 = 1/9, *v* = 5000, 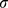 = 0.75, 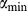 = 0.25, 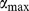 = 0.25, *b* = 500, *c* = 0.1, 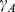 = 0.007, 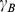 = 0.018, and 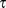 = 12. In (a) *c* = 0.1 and in (b) *m* = 0.42. The line styles are the same as Figure 2.

These behaviours are summarized in Figure 3a, which is a plot of steady state populations against death rate where all other parameters are fixed, and Figure 3b which plots populations and food against *c* the rate of collection of food by foragers.

**Figure 4:**
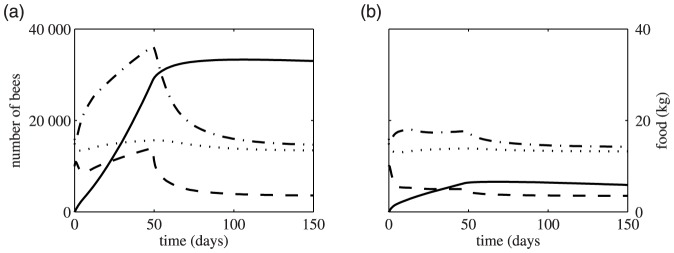
The effect of suddenly increased death rates. Parameter values are as for Figure 2. The solid lines represents food, the dotted lines brood, the dash-dot lines hive bees and the dashed lines foragers. When *t* = 50, the death rate *m* is reset from its initial value to 0.42. In (*a*) the initial death rate is *m* = 0.1 and in (*b*) the initial death rate is *m* = 0.3.. In both plots the hive starts with 16000 hive bees, 10000 foragers, 15000 uncapped brood items and no food at *t* = 0.

Figure 4 shows results from the model when the death rate of foragers is changed suddenly from a value where food stores are increasing to a death rate where food collection will limit hive growth. In Figure 4a, the initial death rate is low, food is accumulating rapidly and the population of adult bees is large and growing. Increasing the death rate leads to a rapid decline in adult bee numbers while food stores cease to grow and start to decline slightly. If the death rate is already high, but not so high that food is limiting (Figure 4b), increasing death rate to a point at which it limits growth results in a much smaller decline in the adult bee population of the hive, and food stores go from growing slowly to declining slowly. In both cases the amount of uncapped brood does not change significantly.

The model gives reasonable agreement with experimental data from Harbo [16], especially for large colonies (Table 1). However, according to Harbo's [16] observations small colonies have much lower food stores (expressed as honey gain per bee per day) than are predicted from our model. There could be many reasons for this. Small colonies may expend more energy per bee for thermoregulation, or may be less efficient at collecting food since a small forage force will be less efficient at identifying rich forage sources in the environment.

**Table 1:** Comparison of model results with observations.

Initial adult bee number		Final brood cell numbers		Brood cells per adult bee		Honey gain per bee per day (mg)		Percentage survival at 22 days	
observed	model	observed	model	observed	model	observed	model	observed	model
2316	2400	4325	4005	2.41	2.13	2.6	23.9	56	56
4515	4500	11162	9154	3.04	2.55	1.6	17.7	64	60
9352	9000	16275	17542	2.21	2.40	10.1	14.7	58	62
17099	18000	22875	26493	1.67	1.79	11.9	15.4	63	64
37061	36000	27875	33599	0.97	1.13	17.7	17.4	55	65

Experimental data is from Harbo, (1986) for hives set up in April. The model results were obtained by running the model for 21 days. The experimental hive was set up for 22 days but the queen was only free to lay after day 2. At the start of each model run, there was no brood or food in the hive and one third of the adult bees were foragers. The parameters used were *L* = 2000, 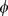 = 1/9, *v* = 5000, 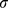 = 1.3, 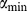 = 0.25, 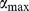 = 0.25, *b* = 500, *c* = 0.09, 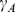 = 0.007, 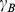 = 0.018, *m* = 0.06 and 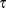 = 21 (which prevented any adult bees emerging during the simulations to match the experimental set-up).

## Discussion

The model that we have presented here is not an attempt to simulate reality; rather the intention of this model is to provide a framework with which to consider the factors that influence colony growth and development, and how they might interact. We have focused on food availability and how that interacts with the intrinsic demographic processes within the colony to affect colony health and growth.

The model suggests that both food availability and forager death rate have very strong influences on colony growth and development. When forager death rates are low, low food availability limits both the amount of food accumulated by the colony, and colony population size (Figure 2b). However as food availability increases, the amount of stored food and total hive population both increase (Figure 3b). Colony population eventually stabilizes at an equilibrium size determined by the forager death rate *m*, and is no longer affected by increasing food availability, whereas food stores continued to increase (Figure 3b). Under conditions of low mortality and high food availability our model predicts an infinite amount of stored food in a colony. Obviously this does not capture the reality of an operating bee colony, but it does reflect to a degree a beekeeper's ideal situation where a colony has the capacity to accumulate a large surplus of honey that can be harvested without compromising colony function.

Varying the death rate has more complex effects on hive dynamics, and as we increase death rates from low to high levels our model produces different categories of equilibrium colony conditions (Figure 3a). Increasing forager death rates from low to moderate levels reduces the equilibrium population size (Figure 2a and 2b). Colonies can still accumulate a surplus of food, although the rate of food accumulation is reduced in smaller colonies (Figure 2a and 2b). At higher death rates, while a colony can maintain a stable, if small, population it is incapable of accumulating a surplus of food. Rather it maintains a small equilibrium food store reflecting a fine balance of food collection and food consumption by the colony (Figure 2c). The population dwindles to zero at death rates exceeding the threshold at which a colony can maintain a stable population, but food stores remain because colonies fail before they have completely consumed their food reserves (Figure 2d). Therefore the model suggests that different forager death rates result in qualitatively and quantitatively different colony outcomes, which range from a stable population with an excess of food stores, to a stable population with limited food stores, to zero population with residual food stores.

This model suggests a hypothesis for the puzzling observation of colonies dying and completely depopulating but leaving a residual food store. Such a situation has been observed with increasing frequency, and has been considered one of the more perplexing features of colony collapse disorder [19], [20]. Our model suggests that depopulation with a small amount of residual food would be expected if colonies suffer a sustained high level of forager mortality (Figure 2d), which is entirely consistent with the rapid declines seen in colony collapse disorder and the lack of dead bees found in the vicinity of a hive [19], [20], [21]. It is unlikely our simple model accurately captures the dynamics of the terminal phase of a colony. When colony populations get small a host of factors could come into play that would accelerate the colony's death, including inability to incubate brood or maintain nest temperature [15], inability to control nest parasites and compromised food collection and processing. These would suggest that colonies would fail far more quickly than is indicated by the model.

The model also indicates the ideal parameter space for maximal honey harvest. In the model low forager mortality coupled with high food availability results in a colony that can continue accumulating a food surplus indefinitely. In reality food stores cannot be infinite but the model does, in some way, represent the fact that healthy bee colonies with abundant forage will continue to accumulate honey reserves daily until they become limited by storage space, seasonal changes or a dearth in forage. It is precisely in this state that beekeepers try to maintain their colonies to produce the greatest possible honey harvest. Our model suggests that to achieve this state, bees should be managed to both maximize forager longevity and capitalize on situations of high food availability. Unfortunately this is easier said than done as the two conditions rarely align for long. Floral resources are transient, and in attempting to maximize foraging opportunities for bees many beekeepers in North America and Europe move their colonies to follow flowering periods of different agricultural crops. But moving a colony imposes a cost of increased forager mortality since experienced foragers are unable to successfully navigate the new environment and become lost [22]. It can take a colony weeks to restore normal forager performance after a move [22]. Therefore the twin ideals of low forager mortality and high food availability are hard to achieve, and some trade off must be made in attempting to optimize colony management. Our model provides a simplified framework for exploring consequences of different approaches to this trade off for colony productivity.

We emphasise that monitoring colony productivity demands attention to food availability, forager longevity and adult bee population. Previously most attention has been paid to food availability, and bee management decisions have been focused on moving colonies to exploit good foraging opportunities, but regardless of food availability colonies will not flourish if forager mortality rates are high. There is increasing interest in development of sensors that could be deployed to aid monitoring of colony performance, and numerous scales, thermometers and humidity sensors have been developed for beekeepers to use in colonies in the field. However, our model indicates that colony weight on its own may not be a good index of colony condition. The lag in the model in the change of overall food stores and brood weight in response to increased forager mortality suggests that colony weight would be a poor early indicator of a dwindling forager population (Figure 4). Examination of the brood nest for the amount of brood may also give a poor indicator of a colony under stress as the model suggests brood responds quite slowly to a change in forager mortality (Figure 4a), and this has been reported for colony deaths related to colony collapse disorder [21].

In our model the nurse bee population declines fastest, and as nurse bees transition to forager bees this partially buffers the decline in forager population (Figure 4). As a consequence simply tracking hive traffic could underestimate the rate at which a colony is depopulating. We suggest that the best strategy for monitoring both level and rate of change of stored food and colony population in bee colonies would require monitoring both change in mass, and the rate of loss of field bees.

There are many obvious ways in which this model could be extended. It does not, for example, take into account seasonal variations in food supply or the queen's egg laying rate, nor does the model distinguish between nectar and pollen collection and consumption. There is always a balance between keeping models simple so that the influences of the most important factors on major outcome can be easily explored and formulating complicated models that include more, but where the overall picture can be harder to grasp. In this paper we have chosen to use a simple model to look at major features of colony demographics in a straightforward way but it would be easy to adapt the ideas of this model into a much more complicated computer simulation model. Such a simulation model would be heavily reliant on accurate parameterization if it were to yield meaningful predictions of how environmental changes might alter colony growth and development. This would demand far more extensive measurement of how colony parameters vary with availability of pollen and nectar in the environment than are currently available.

Several simulation models for honey bee colonies already exist [23], [24], [25]. Most of these have been written on particular computational platforms for particular purposes. For example Makela et al [24] created a model in the LISP programming language to explore the factors that gave Africanised honey bee reproductive superiority over pure-bred European honey bees. Schmikl and Crailsheim [25] used Mathematica to formulate a very complicated model that extends the model of DeGrandi-Hoffman et al [23], in part, by including the effect of division of labour in the hive, modeled using ideas from the Foraging-for-Work theory [26]. This theory of task allocation is less relevant in bees than in ants because bees have a strong age-based component in task allocation [27], [28] and social inhibition is a very important driver of task specialization [27], [29], [30], [31]. In any case, these simulation models are complicated to understand and to construct and most are tailored to particular situations, as reflected in either their intrinsic structure or their parameterization or both. They are very useful when a specific question needs to be addressed and the necessary input data is available, but it is harder to get a large scale, general picture from complex simulation models than from much simpler, less tightly specific differential equation models such as the one that we present here. The type of model that is most useful will depend, to a very large extent, on the purpose of the modeling and on the scale of the relevant dynamics under investigation; that is, whether, for example, details of individual-to-individual interactions are important to the outcome of the model or whether averages can be taken across the populations of classes of bees as in the model presented here.

## Supporting Information

Appendix S1 **Equations for steady states and critical parameter values.**
(PDF)

## References

[1] Khoury DS, Myerscough MR, Barron AB (2011) A quantitative model of honey bee colony population dynamics. PLoS ONE 6: e18491. 21533156 10.1371/journal.pone.0018491 PMC3078911

[2] Neumann P, Carreck NL (2010) Honey bee colony losses. Journal of Apicultural Research 49: 1–6.

[3] Brodschneider R, Crailsheim K (2010) Nutrition and health in honey bees. Apidologie 41: 278–294.

[4] Kearns CA, Inouye DW (1993) Techniques for Pollination Biologists. Boulder CO: University Press of Colorado.

[5] Seeley TD (1995) The Wisdom of the Hive. Cambridge: Harvard University Press.

[6] Toth AL, Robinson GE (2005) Worker nutrition and division of labour in honeybees. Animal Behaviour 69: 427–435.

[7] Schulz DJ, Huang Z-Y, Robinson GE (1998) Effects of colony food shortage on behavioral development in honey bees. Behavioral Ecology and Sociobiology 42: 295–303.

[8] Blaschon B, Guttenberger H, Hrassnigg N, Crailsheim K (1999) Impact of bad weather on the development of the broodnest and pollen stores in a honeybee colony (Hymenoptera : Apidae). Entomologia Generalis 24: 49–60.

[9] Schmickl T, Crailsheim K (2001) Cannibalism and early capping: strategy of honeybee colonies in times of experimental pollen shortages. Journal of comparative physiology A, Sensory, neural, and behavioral physiology 187: 541–547. 10.1007/s003590100226 11730301

[10] Schmickl T, Crailsheim K (2002) How honeybees (*Apis mellifera* L.) change their broodcare behaviour in response to non-foraging conditions and poor pollen conditions. Behavioral Ecology and Sociobiology 51: 415–425.

[11] Jones J, Helliwell P, Beekman M, Maleszka R, Oldroyd B (2005) The effects of rearing temperature on developmental stability and learning and memory in the honey bee, *Apis mellifera* . Journal of Comparative Physiology A 191: 1121–1129. 10.1007/s00359-005-0035-z 16049697

[12] Fahrbach SE, Robinson GE (1996) Juvenile hormone, behavioral maturation and brain structure in the honey bee. Developmental Neuroscience 18: 102–114. 8840089 10.1159/000111474

[13] Robinson GE, Page RE, Strambi C, Strambi A (1992) Colony integration in honey bees: mechanisms of behavioural reversion. Ethology 90: 336–350.

[14] Becher M, Hildenbrandt H, Hemelrijk C, Moritz R (2010) Brood temperature, task division and colony survival in honeybees: a model. Ecological Modelling 221: 769–776.

[15] Rosenkranz P (2008) Report of the Landesanstalt für Bienenkunde der Universität Hohenheim for the year 2007. Stuttgart-Hohenheim, Germany.

[16] Harbo JR (1986) Effect of population size on brood production, worker survival and honey gain in colonies of honeybees. Journal of Apicultural Research 25: 22–29.

[17] Harbo JR (1993) Effect of brood rearing on honey consumption and the survival of worker honey bees. Journal of Apicultural Research 32: 11–17.

[18] Russell R, Barron AB, Harris D (2013) Dynamic modelling of honey bee (Apis mellifera) colony growth and failure. Ecological Modelling In press.

[19] VanEngelsdorp D, Evans JD, Saegerman C, Mullin C, Haubruge E, et al (2009) Colony Collapse Disorder: A Descriptive Study. PLoS ONE 4 10.1371/journal.pone.0006481 PMC2715894 19649264

[20] Watanabe ME (2008) Colony collapse disorder: Many suspects, no smoking gun. Bioscience 58: 384–388.

[21] Oldroyd BP (2007) What's killing American honey bees? PLoS Biology 5: 1195–1199. 10.1371/journal.pbio.0050168 PMC1892840 17564497

[22] Lindauer M (1961) Communication Among Social Bees; Mayr E, Thimann KV, Griffin DR, editors. Cambridge Massachusetts: Harvard University Press.

[23] DeGrandi-Hoffman G, Roth SA, Loper GL, Erickson EH Jr (1989) Beepop: a honeybee population dynamics simulation model. Ecological Modelling 45: 133–150.

[24] Makela ME, Rowell GA, Sames WJ IV, Wilson LT (1993) An object-oriented intracolonial and population level model of honey bees based on behaviors of European and Africanized subspecies. Ecological Modelling 67: 259–284.

[25] Schmickl T, Crailsheim K (2007) HoPoMo: A model of honeybee intracolonial population dynamics and resource management. Ecological Modelling 204: 219–245.

[26] Franks NR, Tofts C (1994) Foraging for work - how tasks allocate workers. Animal Behaviour 48: 470–472.

[27] Beshers SN, Fewell JH (2001) Models of division of labor in social insect colonies. Annual Review of Entomology 46: 413–440. 10.1146/annurev.ento.46.1.413 11112175

[28] Huang Z-Y, Robinson GE (1996) Regulation of honey bee division of labor by colony age demography. Behavioral Ecology and Sociobiology 39: 147–158.

[29] Robson SK, Beshers SN (1997) Division of labour and ‘foraging for work’: simulating reality versus the reality of simulations. Animal Behaviour 53: 214–218.

[30] Leoncini I, Le Conte Y, Costagliola G, Plettner E, Toth AL, et al (2004) Regulation of behavioral maturation by a primer pheromone produced by adult worker honey bees. Proceedings of the National Academy of Sciences of the United States of America 101: 17559–17564. 15572455 10.1073/pnas.0407652101 PMC536028

[31] Huang Z-Y, Robinson GE (1999) Social control of division of labor in honey bee colonies. In: Detrain C, Deneubourg JL, Pasteels JM, editors. Information Processing in Social Insects. Basel: Birkhauser. pp. 165–187.

