# Supplementary material for: Modelling Food and Population Dynamics in Honey Bee Colonies
Source: PLoS One. 2013 May 7;8(5):e59084. doi: 10.1371/journal.pone.0059084 (PMC3647073; doi:10.1371/journal.pone.0059084)
Supplement: Appendix S1 — Equations for steady states and critical parameter values. (PDF) [file pone.0059084.s001.pdf]

Appendix: Equations for steady states and critical parameter values.

When food increases indefinitely (that is, $f \to \infty$ as $t \to \infty$) then $f^2/(f^2+b^2) \approx 1$ and $b^2/(f^2+b^2) \approx 0$ so that the equations for the rates of change of the populations of brood, hive bees and foragers, respectively are approximated by

$$\frac{dB}{dt} = L\frac{H}{H+v} - \phi B \qquad \text{(A1)}$$

$$\frac{dH}{dt} = \phi B(t-\tau) - H\left(\alpha_{\min} - \sigma\frac{F}{F+H}\right) \qquad \text{(A2)}$$

$$\frac{dF}{dt} = H\left(\alpha_{\min} - \sigma\frac{F}{F+H}\right) - mF. \qquad \text{(A3)}$$

(See also equation (8) in the main text.) When (A1), (A2) and (A3) are at steady state then:

$$F = \frac{LQ - mv}{mQ}, \quad H = QF \text{ and } B = \frac{m}{\phi}F \qquad \text{(A4)}$$

where Q is the larger solution of the following quadratic equation:

$$\alpha_{\min}Q^2 + (\alpha_{\min} - \sigma - m)Q - m = 0. \qquad \text{(A5)}$$

When food does not increase indefinitely but approaches a steady state value then the steady state are the solutions of equations (1), (3), (4) and (7) using functions (2) and (5) when $\frac{dB}{dt} = \frac{dH}{dt} = \frac{dF}{dt} = \frac{df}{dt} = 0$. The steady state values for food stores, and forager, hive bee and brood populations are:

$$f = b\sqrt{\frac{\alpha_{\max}}{\frac{\sigma}{P+1} - \frac{m}{P} - \alpha_{\min}} - 1}, \quad F = \frac{Lf^2}{m(f^2+b^2)} - \frac{v}{P}, \quad H = PF \text{ and } B = \frac{m}{\phi}F \qquad \text{(A6)}$$

where

$$P = \frac{c}{\gamma_A} - 1 - \frac{\gamma_B m}{\gamma_A \phi}. \qquad \text{(A7)}$$

The hive goes extinct at the critical death rate

$$m = \frac{\phi\gamma_A}{\gamma_B}\left(\frac{c}{\gamma_A} - 1\right) \qquad \text{(A8)}$$

where P becomes negative. Food ceases to be limiting and goes to infinity at steady state when the denominator in the steady state expression for f in (A6) goes to zero; that is when $\frac{\sigma}{P+1} - \frac{m}{P} - \alpha_{\min} = 0$. This occurs when m is a root of the equation

$$\left(\frac{\gamma_B}{\gamma_A\phi}\right)m^2 - \left(\frac{\sigma\gamma_B}{\gamma_A\phi} - \frac{c}{\gamma_A}\right)m + \alpha_{\min} + \alpha_{\max} + \sigma - \frac{\sigma c}{\gamma_A} = 0. \qquad \text{(A6)}$$
